# Supplementary material for: Molecular Profiling of A549 Cell-Derived Exosomes: Proteomic, miRNA, and Interactome Analysis for Identifying Potential Key Regulators in Lung Cancer
Source: Cancers (Basel). 2024 Dec 10;16(24):4123. doi: 10.3390/cancers16244123 (PMC11674491; doi:10.3390/cancers16244123)
Supplement: Supplementary file 1 [file cancers-16-04123-s001.zip › cancers-3335801-supplementary.pdf]

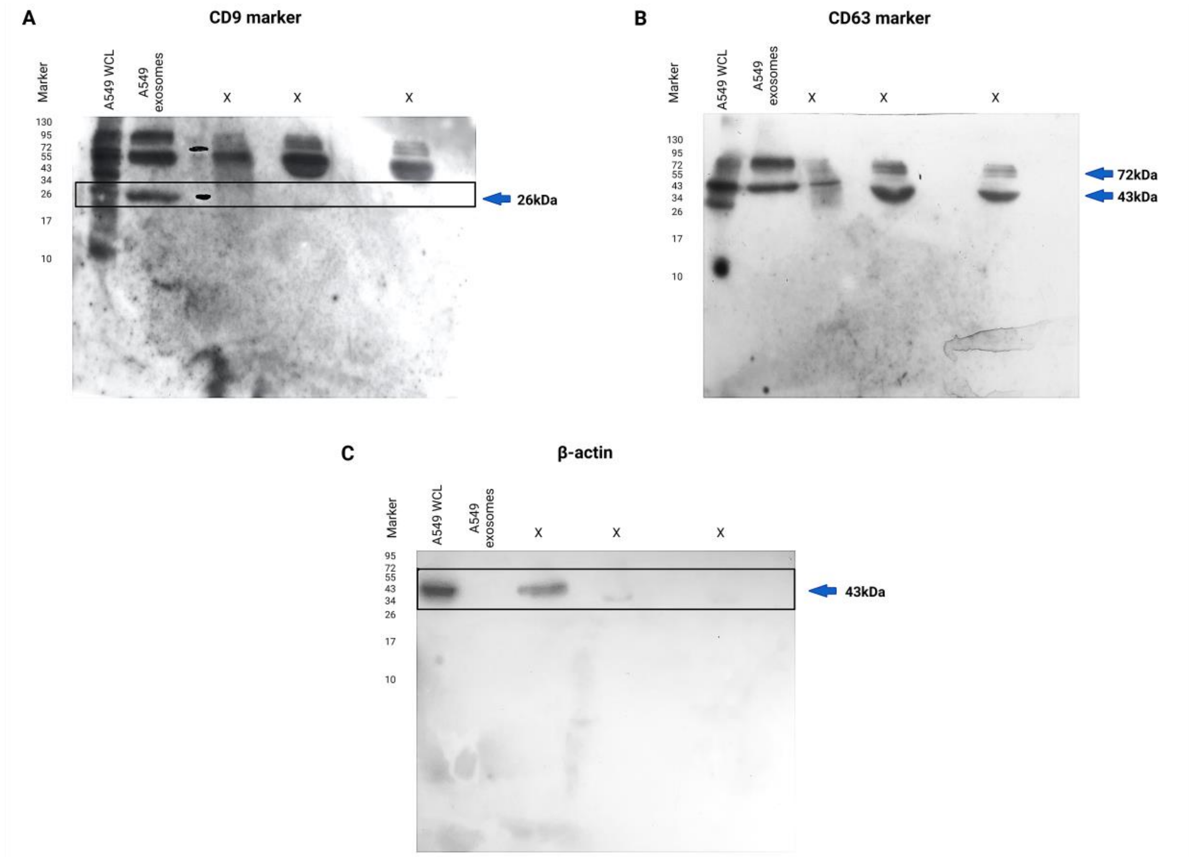


***Supplementary Figure S1- Western blotting evaluation of CD9, CD63 and β-actin expression in A549 Whole Cell Lysate and A549 cell-derived exosomes.*** *Equal amount of protein (30μg) from exosomes and whole cell lysate as calculated by BCA protein assay kit were loaded in wells of 15% SDS-PAGE gel for separation, followed by Western Blotting. CD9 (A) and CD63 (B) protein marker were detected in both WCL and A549 exosomes. (C) The expression of β-actin was detected only in WCL,supporting minimal cytoplasmic contamination in exosomal samples. Chemiluminescent signals were visualized using Immobilon ECL Ultra Western HRP Substrate and exposed to X-ray film for detection. The developed films were subsequently digitized using a gel imaging system.*
